# Empowering visually impaired students through innovative tools and accessible waste sorting education at the national level

**DOI:** 10.1371/journal.pone.0323171

**Published:** 2025-05-07

**Authors:** Patranit Srijuntrapun, Issavara Sirirungruang, Chawalit Nucharoen

**Affiliations:** 1 Department of Education, Faculty of Social Sciences and Humanities, Nakhon Pathom, Mahidol University, Thailand; 2 Faculty of Medicine Ramathibodi Hospital, Ratchasuda Institute, Mahidol University, Nakhon Pathom, Thailand; 3 Faculty of Social Sciences and Humanities, Mahidol University, Nakhon Pathom, Thailand; University of Washington, UNITED STATES OF AMERICA

## Abstract

Waste separation is a critical strategy to address the intensifying global waste crisis. However, inclusive teaching strategies for visually impaired students remain limited. This study addresses this pressing gap by implementing an innovative intervention rooted in experiential learning principles. This approach integrates sound-equipped bins and waste sorting activity guides tailored to empower visually impaired students to independently and effectively separate waste. The research study employed a mixed-methods design encompassing three key phases: (1) training teachers to proficiently use accessible tools and to deliver comprehensive waste management education, (2) training visually impaired students through hands-on experiential learning to develop independent waste separation skills, and (3) evaluating behavior changes and skill retention among students after the training. The results demonstrated significant statistical improvements in knowledge (mean increase = 0.74, p < 0.001), attitudes (mean increase = 0.50, p < 0.001), and practical skills (mean increase = 0.55, p < 0.001) by students. These findings highlight the transformative potential of inclusive educational innovations to bridge existing gaps and promote self-reliance in waste separation among visually impaired learners. The study emphasizes the importance of adopting creative, evidence-based strategies to ensure inclusivity and sustainability in environmental education on a global scale. Policymakers and educators are urged to leverage these insights to advance environmental literacy and foster equitable participation in waste management practices.

## Introduction

In the world, approximately 2.1 billion tons of municipal solid waste are annually generated, with at least 38% (810 million tons) being improperly managed [1]. This poses significant threats to environmental health in the form of pollution [2] and greenhouse gas emissions [3]. Waste separation is essential for mitigating these impacts, enabling the recycling of materials and the conservation of natural resources [4,5]. However, efforts to promote inclusive waste management practices remain insufficient for individuals with disabilities, particularly those who are blind or visually impaired. According to a 2020 report from the International Agency for the Prevention of Blindness (IAPB), 43 million people globally are blind, while 295 million experience moderate to severe visual impairment [6]. In Thailand, approximately 170,955 individuals are blind. These individuals face significant challenges, including barriers to education and employment opportunities [7], which further limit their participation in waste separation initiatives [8]. Because of these restrictions, their engagement in sustainability efforts is insufficient, underscoring the need for inclusive waste separation strategies that accommodate diverse abilities, as a way to protect public health and promote equity in environmental stewardship [9].

Like many developing countries, Thailand is facing a growing waste management crisis. In 2023, the country generated approximately 26.95 million tons of municipal solid waste [10]. Most of this waste was mismanaged, leading to environmental and public health concerns. Improper disposal of face masks, a crucial tool during the COVID-19 pandemic, has exacerbated waste management challenges. These challenges are shared by many developing countries [11], and even in developed nations like the United States the pandemic has disrupted waste processing systems [12]. Lockdowns and economic slowdowns disproportionately affected marginalized communities. Informal waste collectors, for example, faced income loss and increased health risks due to improperly disposed waste related to the pandemic. In Nigeria, policy gaps left informal waste workers vulnerable to rising waste accumulation [13], while fragile infrastructures were strained by the increase in hazardous medical and plastic waste, leading to environmental contamination [14]. These disparities underscore the urgent need for inclusive policies that integrate marginalized groups into formal waste governance.

Thailand’s waste management policies have many things in common with those of other developing nations, such as Nigeria, Uganda, and Benin [15–18]. But they also exhibit distinct characteristics. Thailand employs a multifaceted approach that integrates policy-driven interventions, including the Action Plan on National Waste Management, Phase 2 (2022–2027) [19], which is strategically designed to align with the Sustainable Development Goals (SDGs) and the National Economic and Social Development Plan. This policy framework emphasizes resource efficiency and sustainable waste management through certain key principles, including the Bio-Circular-Green Economy (BCG Model), the 3R approach (Reduce, Reuse, Recycle), the Polluter Pays Principle (PPP), Public-Private Partnerships (PPP), and Extended Producer Responsibility (EPR). A key component of this strategy is Thailand’s commitment to Education for Sustainable Development (ESD), which has been systematically incorporated into national education policies through the framework of the Sufficiency Economy Philosophy (SEP) [20]. This approach aligns with waste management projects in Nigeria and Uganda, where public awareness campaigns and grassroots participation have been instrumental in enhancing waste management efficiency despite the limitations in infrastructure [16,21]. In contrast to Thailand’s integrated model, many developing countries continue to focus predominantly on infrastructure development as the primary means of shaping their waste management systems, often overlooking the critical role of education and behavioral change in achieving long-term sustainability [22,23].

Despite extensive research on waste management education and the promotion of inclusivity in educational institutions [24–26], a critical gap exists in the integration of these domains, particularly regarding the waste segregation education of visually impaired students. While teaching waste segregation to sighted students is a well-established practice [27–29], educational approaches specific for visually impaired students remain underdeveloped. Incorporating waste segregation practices into educational initiatives aimed at individuals with disabilities is pivotal for fostering a sustainable society in alignment with the United Nations Sustainable Development Goals (SDGs), specifically SDG 11 (Sustainable Cities and Communities) and SDG 12 (Responsible Consumption and Production) [1]. Such integration empowers students with disabilities to build environmental and social responsibility. Specialized tools and methodologies are essential for creating an inclusive learning environment for visually impaired students that supports independent and accurate waste segregation [30–32].

In Thailand, where 16 schools for the blind serve visually impaired students, there are no targeted programs or tools specifically designed to teach them effective waste segregation. Current practices are limited to lectures on waste separation. Conventional methods, such as color-coded bins, are ineffective for visually impaired students as they rely predominantly on visual cues, rendering them inaccessible. Additionally, experiential learning methods emphasizing hands-on participation are rarely implemented, highlighting a significant gap in developing and deploying inclusive waste management interventions for visually impaired individuals [33]. This leads to the research question: Can experiential learning using specially designed bins for visually impaired individuals enhance their waste segregation skills and sustain lasting behavioral changes?

This study aims to evaluate the application of experiential learning combined with innovative sound-equipped bins designed specifically for visually impaired students. A training program was implemented at both the teacher and student levels, enabling visually impaired students to effectively and independently separate waste. The novelty of this study lies in the pioneering application of sound-equipped bins ([34] as an innovative intervention tool that enhances waste management accessibility for visually impaired individuals. Unlike traditional systems that rely on visual differentiation, these bins use audio cues to facilitate experiential learning, enabling visually impaired students to independently sort waste. Their implementation in 11 out of 16 schools for the blind in Thailand underscores their real-world feasibility and potential for nationwide scalability. This cost-effective and adaptable approach creates a lasting impact by equipping generations of visually impaired students with sustainable waste management skills, in alignment with the United Nations Sustainable Development Goals (SDGs). By embedding accessible waste management strategies into educational policies, this study provides a model that integrates accessibility-focused sustainability initiatives and encourages policymakers around the world to adopt evidence-based and inclusive frameworks for environmental education.

## Enhancing experiential learning for students with visual impairments

The notion of experiential learning has predominantly been developed under the framework of Experiential Learning Theory (ELT) proposed by Kolb [35]. According to Kolb & Kolb [36], experiential learning is a process where knowledge is constructed through experience. Unlike traditional forms of learning, this approach actively engages learners in the process of acquiring knowledge and skills through hands-on experience. The learning cycle of ELT comprises four distinct stages:

1) Concrete Experience: This stage involves learners actively engaging in real-life activities or experiences. It fosters learning through direct interaction, emphasizing the sensory and emotional dimensions of actual experiences.
2) Reflective Observation: At this stage, learners thoughtfully observe and reflect on their experiences. This reflection helps them gain deeper insights and make sense of what has occurred.
3) Abstract Conceptualization: During this phase, learners synthesize their reflections and observations into abstract concepts or theories, forming their understanding and constructing new knowledge frameworks.
4) Active Experimentation: Finally, learners apply their conceptualized ideas in various contexts, testing their understanding and refining their approaches. This application promotes the creation of new experiences, thus completing and renewing the experiential learning cycle.

This cycle, where learners actively construct knowledge through iterative practice and reflection, underscores the dynamic and participatory nature of experiential learning. This adaptable framework transforms traditional education by actively engaging learners and optimizing information delivery methods. Educators initiate the learning process through experiential activities, followed by individual or group reflection. The conceptualization phase deepens understanding through lectures or relevant readings, while the application phase encourages learners to integrate their insights into personal or professional contexts [37].

The experiential learning (EL) cycle has been widely implemented across educational settings, from individual classes and training programs to entire curricula and national education policies [38]. Four key educator roles are integral in guiding students through the EL cycle: (1) the *facilitator* assists students in learning from their personal experiences while fostering their reflection, (2) the *subject expert* provides reliable knowledge and organizes reflective thoughts into structured concepts, (3) the *standard-setter* or *evaluator* establishes performance standards and evaluates the practical application of knowledge, and (4) the *coach* encourages learners to apply knowledge in real-world scenarios, offering one-on-one support.

Experiential learning enhances learner engagement, motivation, confidence, and effectiveness, contributing positively to skill development in the classroom [39]. Kolb and Kolb [36] emphasize that EL ensures that individuals benefit from experience in ways that enhance the learning outcomes more effectively than in traditional environments. This approach not only increases knowledge retention but also fosters discipline-specific competencies. By integrating EL into diverse educational frameworks, institutions can cultivate active, reflective, and adaptive learners prepared to apply their knowledge in meaningful ways [33].

Experiential learning has long been recognized for its extensive educational benefits across a variety of fields [40]. It has been widely adopted in post-secondary education [41,42], with applications in online education [43], art and design [44], medical healthcare [45], and political science [46,47]. Furthermore, its relevance extends to the active promotion of experiential activities such as role-playing, simulations, and hands-on experiments, particularly those aimed at deepening the understanding of disabilities [33,48–50].

Despite growing pedagogical interest in EL, research on how to apply this theory and its methods to students with disabilities remains limited. In this context, one particularly underrepresented group are those students with “invisible disabilities” [33]. Blind students often face significant exclusion, driven in part by the limited availability of teacher support and the prevailing perceptions of visual impairment as a deficiency [51]. Furthermore, existing instructional materials frequently fail to accommodate the specific needs of visually impaired learners.

To address these challenges, the concept of Universal Design (UD) has been adapted to create inclusive teaching materials for this study. The UD framework is grounded on the principle that each learner has unique characteristics and requirements [52]. Universal Design for Learning (UDL) expands this approach by emphasizing accessible environments and accommodations, enabling students to fully realize their potential. UDL focuses on creating equitable learning opportunities through accessible resources, adaptive environments, and supportive measures. In higher education, institutions increasingly provide accommodations such as accessible facilities and assistive learning tools to ensure equal opportunities for all students [53].

## Methodology

There are approximately 200,000 visually impaired individuals in Thailand and the country has 16 specialized schools for the blind. These schools operate under a policy framework designed to provide equal and inclusive learning opportunities for everyone with the support of the Educational Promotion and Development Fund for Handicapped Groups. This fund, established under The Persons with Disabilities Education Act B.E. 2551 (2008), aims to ensure comprehensive and equitable access to education for children with disabilities. Specialized schools and inclusive programs offer tailored environments for blind children, using assistive technologies, Braille materials, and accessibility adaptations. The fund provides financial aid for institutions, training for educators, and community engagement to promote quality education and social integration [54].

### Study design

This study employed a mixed-methods approach, combining both quantitative and qualitative data collection methods to comprehensively evaluate the impact of an intervention program aimed at improving waste segregation practices among visually impaired students. The data was collected from specialized schools for the blind across Thailand between 1 March and 31 August 2022.

*Quantitative data* consisted of pre- and post-training assessments that measured students’ knowledge, attitudes, and skills related to waste segregation. This data was collected using structured questionnaires designed to measure changes in behavior and understanding as a result of the intervention. *Qualitative data* was gathered through semi-structured interviews with teachers and observations of students during experiential learning sessions. This data provided insights into the experiences of participants with the intervention tools (e.g., sound-equipped bins) as well as into their perceptions regarding the effectiveness of these tools. Additionally, qualitative data helped to contextualize the behavioral changes observed during follow-up evaluations. This combination of quantitative metrics and qualitative narratives allowed for a robust evaluation of both the immediate outcomes and the long-term sustainability of waste segregation practices among visually impaired students.

The study was structured in three phases. 1) Teacher training: The first phase involved the training of teachers, with the aim of providing them with the knowledge and skills necessary to use intervention tools, including innovative sound-equipped bins and waste sorting activity guides. This training enabled them to effectively teach waste segregation principles to visually impaired students. 2) Student training through experiential learning sessions: In the second phase, the trained teachers implemented experiential learning sessions with students, focusing on the use of sound-equipped bins to facilitate efficient waste segregation and foster independent waste separation skills. 3) Behavioral assessment: The final phase assessed changes in waste segregation behavior among blind students, evaluating the sustainability of their practices one month after receiving training.

### Intervention tools

This study utilized “sound-equipped bin” specifically designed to assist visually impaired students in accurately separating waste. These innovative waste bins are registered under patent number 17547 [55].

The “waste sorting guide for the blind” served as an intervention tool designed to assist teachers in facilitating waste segregation activities for visually impaired students. The guide was available in both standard and Braille formats. It comprised five practical activities: (1) knowledge about different types of waste, (2) specific knowledge about face mask waste, (3) impacts of general and face mask waste, (4) correct methods for separating face masks and other types of waste, and (5) preparation of face mask waste for disposal. Each session included detailed content on objectives, time allocation, required materials, and step-by-step activity guidelines, fostering a positive and inclusive learning environment for students.

Both the sound-equipped bins and the waste sorting activity guides for blinds were integral to the training sessions. They were used in practical demonstrations to enhance learners’ understanding and skill.

### Data collection and characteristics of participants

During phase 1, conducted in 2022 in the midst of the COVID-19 pandemic, teachers were trained online. The training was specifically designed for teachers working in schools for the blind and encompassed several key components: general knowledge about face masks and other categories of waste, their environmental impacts, and proper waste segregation methods. Practical demonstrations of the sound-equipped bins were also part of the training, which used the waste segregation manuals in both standard and Braille formats. Teachers were also trained in the basic maintenance of the sound-equipped bins. Each target school received sound-equipped bins prior to the training, providing hands-on experience with these tools and laying the groundwork for subsequent student training. To evaluate the effectiveness of this intervention, pre-test and post-test assessments were conducted, measuring changes in knowledge, attitudes, and skills among participants.

For inclusion criteria, the participants were all teachers of visually impaired students selected through purposive sampling. A total of 20 teachers selected from 11 schools for the blind took part in the training. Participants were required to have a minimum of three years of experience working with visually impaired students to ensure that they had expertise and familiarity with their needs and challenges. All participants expressed their willingness to engage with the research project.

During phase 2, visually impaired students were the target of training aimed at helping them transition from guided learning to independently applying waste segregation skills. Teachers followed the waste sorting activity guides for blinds, providing real-time feedback and support until students could accurately use the sound-equipped bins. Additional assistance was offered as needed. To sustain independent waste segregation, the sound-equipped bins were installed in easily reachable areas within the schools. Pre-test and post-test assessments were conducted to evaluate the impact of the intervention on the knowledge, attitudes, and skills of students.

The participants were all visually impaired students from the 11 schools, selected via purposive sampling. The inclusion criteria required students to be capable of fully participating in the training program. Students with severe additional disabilities or those who declined to participate were excluded based on the established criteria. The training program engaged a total of 457 student participants.

Phase 3 focused on evaluating the retention of waste segregation behaviors among the visually impaired students that participated in the program. This involved monitoring their progress and behavior one month after the waste segregation training had been completed. Teachers conducted observational assessments to track and document the students’ ability to sustain the practices that they had learned during the training program.

The participants in this phase were representative teachers from the 11 schools for the blind selected for the study. Using purposive sampling, one teacher was selected from each of the schools. Selection criteria required that the teachers had participated in phase 1 and be willing to observe and evaluate the behavior of students. In total, 11 teachers were included in this phase of the study. This approach provided insights into the long-term effectiveness of the intervention and the sustainability of the waste segregation skills acquired by students.

### Measurements

In phases 1 and 2, pre- and post-intervention assessments were conducted to evaluate changes in waste segregation behavior, focusing on three key variables: knowledge, attitudes, and skills. Knowledge about how to manage face mask waste during the COVID-19 pandemic was measured using eight dichotomous questions, with correct responses scored as 1 and incorrect responses as 0. Attitudes toward waste management and the segregation of disposable face masks were assessed using a four-item survey over a three-point Likert scale (1 = low, 2 = moderate, 3 = high). Finally, skill in the use of sound-equipped bins for managing and segregating face mask waste was evaluated through surveys consisting of four items for teachers and two items for students. These were rated over a five-point Likert scale (1 = least skills, 5 = Highest skills). To ensure that the content was valid and appropriate, the questionnaire was evaluated by three experts, two of them specialized in visually impaired individuals and one in waste management. Reliability was determined using Cronbach’s alpha. A Cronbach’s alpha value between 0.7 and 0.8 is generally considered acceptable in social science research [56]. The resulting value of 0.823 confirmed the instrument’s reliability and suitability for the study.

For phase 3, the retention of waste segregation behaviors among blind students was evaluated through teacher observations and in-depth interviews conducted during the first month after the training. This assessment used a survey with open-ended questions featuring five key questions: 1) application of acquired knowledge in practical settings, 2) adherence to waste segregation and the disposal of face masks and other types of waste in sound-equipped bins, 3) awareness of the importance of waste segregation, 4) recognition of the benefits of segregating and properly disposing of face masks and other types of waste, and 5) engagement in discussions or knowledge-sharing about waste segregation with peers or teachers.

### Data analysis

Quantitative data was analyzed using descriptive statistics, including frequency, percentage, mean, and standard deviation, to summarize the characteristics of the sample. These metrics provided insights into the distribution of the responses and facilitated a clear presentation of the data. To assess the effectiveness of the training interventions, paired t-tests were conducted, comparing participants’ knowledge, attitude, and skill levels before and after the training. This comparison enabled researchers to determine whether the training had resulted in significant improvements. Statistical analysis was performed using SPSS version 25.0 (IBM Corporation).

The qualitative data gathered from semi-structured interviews was examined using content analysis, with particular attention given to the interpretation of the context of post-training behavior. Data coding was based on themes identified from the interviews and derived through iterative readings of notes and translations reflecting participants’ thoughts. Triangulation of multiple data sources, including interviews, reports, and observations, was applied to enhance the reliability of the collected data, following generally accepted qualitative research methodologies [57,58].

### Ethical statement

This research project received ethical approval from the Mahidol University Ethics Committee, under reference number 2021/143.0401. Before commencing data collection, all participants provided written informed consent. The capacity to provide consent was determined by ensuring that all participants were adults (aged above 18 years). For students with visual impairments, written informed consent from a parent or guardian was also required. For students with visual impairments participating in the study, written informed consent from a parent or guardian was also required. They were thoroughly briefed on the study’s objectives, their rights to participate or withdraw, and the confidentiality of their personal information. The Mahidol University Ethics Committee reviewed and approved this consent procedure. Participants were assured that all the collected data would be used strictly for research purposes.

## Results

### Evaluation of teacher training: Knowledge, attitudes, and skills

The effects of the training intervention on teachers’ knowledge regarding the management of face masks and other types of waste were evaluated. The study revealed that, prior to the training, teachers had accurate knowledge regarding plastic and face mask waste, as well as about the impacts of face mask waste. Following the training, knowledge in all areas showed significant improvements, except in the area of disposal, as summarized in Table 1.

**Table 1:** Teachers’ knowledge about the management of general and face mask waste before and after training (n = 20).

Waste Management Knowledge	Correct Answers	
	Before n (%)	After n (%)
1.Snack wrappers are recyclable waste.	4 (20.0)	10 (50.0)
2.Batteries are hazardous waste.	18 (90.0)	19 (95.0)
3.Plastic bottles are recyclable waste.	20 (100.0)	20 (100.0)
4.Face masks used during the COVID-19 pandemic are infectious waste.	20 (100.0)	20 (100.0)
5.Disposed face masks can decompose naturally.	3 (15.0)	18 (90.0)
6.Used face masks and COVID-19 protective equipment may be contaminated with pathogens and pose a risk to others, such as waste collectors.	20 (100.0)	20 (100.0)
7.Leakage of face mask waste into the ocean poses severe risks to marine life.	20 (100.0)	20 (100.0)
8.Face masks used during the COVID-19 pandemic can be disposed with general waste.	16 (80.0)	15 (75.0)

Prior to the training, most participants (70%, n = 18) demonstrated moderate knowledge levels (scores ranging from 2.67 to 5.33), while 30% (n = 2) exhibited high knowledge levels (scores ranging from 5.34 to 8.00). No participants fell into the low knowledge levels (scores ranging from 0.00 to 2.66). Following the training, a substantial improvement was observed in the scores, with 95% of participants achieving high knowledge levels and only 5% remaining in the moderate knowledge category. Notably, no participants exhibited low knowledge levels after training, underscoring the effectiveness of the intervention in enhancing participants’ understanding.

The change in teachers’ attitudes toward the management of face mask waste and other types of waste was also evaluated. The study revealed that, prior to the intervention, teachers exhibited a high level of attitude regarding the benefits of segregating waste, with 85% (n = 17) and 75% (n = 15) acknowledging the benefits of segregating general and face mask waste respectively. Satisfaction with the segregation of general and face mask waste was also notably high, as reported by 55% (n = 11) of participants. After the training, teachers showed an increased awareness of the importance of waste segregation, particularly regarding face mask waste, alongside improved satisfaction levels. High satisfaction regarding general waste segregation was observed in almost 85% (n = 17) of participants, while satisfaction with face mask waste segregation increased to 70% (n = 14). These findings highlight the impact of training on the enhancement of teachers’ attitudes toward effective waste management practices (Table 2).

**Table 2:** Teachers’ attitudes toward the management of general and face mask waste before and after training (n = 20).

Attitude	Before Training				After Training			
	High	Medium	Low	($\overset{―}{x\;}$)	High	Medium	Low	($\overset{―}{x\;}$)
Benefits of general waste segregation for individuals with visual impairments	17 (85.0)	3 (15.0)	0 (0.0)	2.85	17 (85.0)	3 (15.0)	0 (0.0)	2.85
Benefits of segregating face mask waste for individuals with visual impairments	15 (75.0)	5 (25.0)	0 (0.0)	2.75	16 (80.0)	4 (20.0)	0 (0.0)	2.80
Satisfaction with general waste segregation for individuals with visual impairments	11 (55.0)	6 (30.0)	3 (15.0)	2.40	17 (85.0)	3 (5.0)	0 (0.0)	2.85
Satisfaction with face mask waste segregation for individuals with visual impairments	11 (55.0)	7 (35.0)	2 (10.0)	2.45	14 (70.0)	6 (30.0)	0 (0.0)	2.75
Total				2.61				2.80

The study shows that, prior to the training, 80% of respondents exhibited a positive attitude (scores ranging from 2.01 to 3.00), while 20% displayed a neutral attitude (scores ranging from 1.01 to 2.00). None of the respondents had a negative attitude (scores ranging from 0.00 to 1.00). Following the training, 85% of respondents demonstrated a positive attitude, 15% maintained a neutral attitude, and none exhibited a negative attitude.

Finally, teachers’ skills in teaching how to manage general and face mask waste were also evaluated. The study reveals that, prior to the training, 45% of teachers exhibited high-level skills in teaching general waste segregation and disposal to visually impaired students, while 40% demonstrated high-level skills in teaching how to handle face mask waste. Additionally, a significant proportion of teachers displayed moderate skills in conducting activities related to general and face mask waste segregation (40%) as well as in facilitating recreational activities (45%). Assessment after the training indicated that teachers had achieved the highest skill levels across all areas, underscoring the effectiveness of the training program in enhancing both instructional and activity-facilitation skills (Table 3).

**Table 3:** Teachers’ skills in teaching waste segregation before and after training (n = 20).

Skills	Before Training						After Training					
	Highest	High	Moderate	Low	Least	($\overset{―}{x\;}$)	Highest	High	Moderate	Low	Least	($\overset{―}{x\;}$)
Teaching visually impaired students to segregate and dispose of general waste.	4 (20.0)	9 (45.0)	6 (30.0)	1 (5.0)	0 (0.0)	3.80	11 (55.0)	9 (45.0)	0 (0.0)	0 (0.0)	0 (0.0)	4.55
Teaching visually impaired students to segregate and dispose of face mask waste.	4 (20.0)	8 (40.0)	7 (35.0)	1 (5.0)	0 (0.0)	3.75	10 (50.0)	7 (35.0)	3 (15.0)	0 0.0)	0 (0.0)	4.35
Providing activities aimed at general and face mask waste segregation.	4 (20.0)	6 (30.0)	8 (40.0)	2 (10.0)	0 (0.0)	3.60	9 (45.0)	9 (45.0)	2 (10.0)	0 (0.0)	0 (0.0)	4.35
Organizing recreational activities to promote general and face mask waste segregation.	4 (20.0)	5 (25.0)	9 (45.0)	2 (10.0)	0 (0.0)	3.55	11 (55.0)	6 (30.0)	3 (15.0)	0 0.0)	0 (0.0)	4.40

The study also found that, prior to the training, 55% of respondents had a high skill level (scores ranging from 3.34 to 5.00), while 45% exhibited moderate skill levels (scores ranging from 1.67 to 3.33). Notably, no respondents were categorized as having low skills (scores ranging from 1.00 to 1.66). Following the training, 95% of respondents achieved high skill levels, 1% maintained moderate skills, and none were classified as having low skills. These results highlight the effectiveness of training in significantly enhancing the participants’ skill levels.

The differences in knowledge, attitude, and skill scores before and after the training were analyzed using t-tests. The knowledge and attitude scores show a statistically significant difference (P < 0.01), while the comparison of the attitude scores shows no statistically significant difference before and after the training (P > 0.001) (Table 4). These findings will be further discussed in the Discussion section.

**Table 4:** T-tests comparing teacher’s knowledge, attitude, and skill scores.

Dimension		Mean	S.D.	Mean Difference	95% CI: Mean Difference		Paired T-tests		
					Upper	Lower	*t*	*df*	*p*
Knowledge	Pre-test	6.05	0.510	0.95	0.63	−1.46	5.29	19	<.01
	Post-test	7.10	0.718						
Attitude	Pre-test	2.61	0.469	0.19	0.49	−0.12	1.26	19	>.001
	Post-test	2.80	0.359						
Skill	Pre-test	3.67	0.807	1.21	1.21	0.25	3.32	19	<.01
	Post-test	4.41	0.608						

### Evaluation of student training: Knowledge, attitudes, and skills

Students’ improvement in knowledge about general and face mask waste management was also evaluated. Prior to the training, it was observed that 447 visually impaired students (97.81%) had accurate knowledge about plastic waste. However, their understanding of face mask waste was more limited. Specifically, 257 students (56.23%) correctly identified discarded masks as infectious waste, while only 159 students (34.80%) understood that masks should not be discarded with general waste. This limited knowledge may be attributed to the novelty of the COVID-19 pandemic and the associated impacts of face mask usage, which were relatively new topics for visually impaired students. Following the training, significant improvements in participants’ knowledge were observed, as detailed in Table 5.

**Table 5:** Students’ knowledge about the management of general and face mask waste before and after training (n = 457).

Knowledge	Correct Answers	
	Before n (%)	After n (%)
Snack wrappers are recyclable waste.	284 (62.14)	449 (98.25)
Batteries are hazardous waste.	334 (73.09)	455 (99.56)
Plastic bottles are recyclable waste.	447 (97.81)	453 (99.12)
During the COVID-19 pandemic, used face masks are infectious waste.	257 (56.23)	368 (80.52)
Face mask waste can decompose naturally.	389 (85.12)	454 (99.34)
Used face masks and COVID-19 protective equipment may be contaminated with pathogens and pose a transmission risk to others, such as waste collectors.	361 (78.99)	454 (99.34)
Leakage of face mask waste into the ocean poses severe risks to marine life.	433 (94.75)	454 (99.34)
During the COVID-19 pandemic, face mask waste can be disposed of with general waste.	159 (34.80)	387 (84.68)

The results shown in Table 5 reveal a substantial improvement in knowledge following the training. Prior to the training, most participants (74.60%) demonstrated a high level of knowledge (scores between 5.43 and 8.00) while 25.40% exhibited a moderate level of knowledge (scores ranging from 2.67 to 5.33). Notably, no participants scored within the low knowledge range (0.00 to 2.66). The results after the training show a dramatic increase in knowledge, with 99.30% of participants achieving a high level of knowledge and only 0.70% remaining at a moderate level, while no participants scored in the low range. This significant improvement underscores the effectiveness of the training intervention.

In addition, students’ attitudes toward general and face mask waste management were also evaluated. Before the intervention, students recognized the benefits of separating general and face mask waste to a certain extent. Regarding general waste, 371 students (81.18%) expressed moderate acknowledgment, while 346 students (75.71%) expressed similar levels of recognition regarding face mask waste. Satisfaction levels were also moderate, with 374 students (81.84%) reporting satisfaction with general waste and 365 students (79.87%) being satisfied with face mask waste separation.

Following the training, students demonstrated an increased perception of the benefits of waste separation. However, the level of satisfaction with general waste separation remained at a moderate level, as reported by 246 students (53.83%). In contrast, satisfaction with face mask waste separation significantly improved, reaching a high level for 294 students (64.99%). These results are summarized in Table 6.

**Table 6:** Students’ attitudes toward face mask waste and other types of waste management before and after training (n = 457).

Attitude	Before Training				After Training			
	High	Medium	High	($\overset{―}{x\;}$)	High	Medium	High	($\overset{―}{x\;}$)
Benefits of general waste segregation for individuals with visual impairments	84 (18.38)	371 (81.18)	2 (0.44)	2.17	366 (80.09)	79 (17.29)	12 (2.62)	2.77
Benefits of face mask waste segregation for individuals with visual impairments	106 (23.20)	346 (75.71)	5 (1.09)	2.22	389 (85.12)	67 (14.66)	1 (0.22)	2.84
Satisfaction with general waste segregation by individuals with visual impairments	64 (14.00)	374 (81.84)	19 (4.16)	2.09	206 (45.08)	246 (53.83)	5 (1.09)	2.43
Satisfaction with face mask waste segregation by individuals with visual impairments	82 (17.94)	365 (79.87)	10 (2.19)	2.15	294 (64.99)	158 (33.92)	5 (1.09)	2.63
Total				2.16				2.67

The results of the study show a significant shift in the attitudes of participants following the training. Before the training, 364 respondents (79.65%) exhibited a moderate attitude (scores ranging from 1.01 to 2.00) while 84 respondents (18.38%) had a positive attitude (scores between 2.01 and 3.00). Only 9 respondents (1.97%) had a negative attitude (scores between 0.00 and 1.00). After the training, 313 respondents (68.50%) displayed a positive attitude, 138 respondents (30.20%) maintained a moderate attitude, and only 6 respondents (1.30%) held a negative attitude. These findings highlight the effectiveness of the training in fostering more positive attitudes among participants.

Finally, students’ skills in using sound-equipped bins were also evaluated. The study revealed that, prior to the training, most visually impaired students had a high level of skill in sorting and disposing of general (196 students, 42.89%) and face mask waste (212 students, 46.39%) into sound-equipped bins. Following the training, the skill level remained high, but there was a notable increase in the number of students achieving the ‘highest’ skill level for both general (97 students, 21.22%) and face masks (189 students, 41.36%) waste disposal. These results, shown in Table 7, underscore the effectiveness of the training program in enhancing waste segregation skills among visually impaired students.

**Table 7:** Students’ skills in using sound-equipped bins before and after training (n = 457).

Skill	Before Training						After Training					
	Highest	High	Moderate	Low	Least	($\overset{―}{x\;}$)	Highest	High	Moderate	Low	Least	($\overset{―}{x\;}$)
Skills in general waste segregation and disposal into designated bins	50 10.94)	196 42.89)	181 39.61)	30 6.56)	0 0.00)	3.58	97 21.22)	245 53.61)	104 22.76)	11 2.41)	0 0.00)	3.93
Skills in face mask waste segregation and disposal into designated bins	42 9.20)	212 46.39)	124 27.13)	69 15.09)	10 2.19)	3.45	189 41.36)	232 50.77)	31 6.78)	5 1.09)	0 0.00)	4.32

As found by the study, prior to the training, 452 respondents (98.91%) had high-level skills (scores between 3.34 and 5.00) while 5 respondents (1.09%) exhibited moderate skills. No participants fell within the low-skill range. Following the training, all respondents (457, 100%) achieved high-level skills, with no individuals exhibiting moderate or low skills. These results underscore the training program’s effectiveness in elevating participants’ skill levels to a uniformly high standard.

The pre-test and post-test differences in total scores for knowledge, attitude, and skill were also analyzed. As summarized in Table 8, this analysis shows a statistically significant difference (*P* < 0.001).

**Table 8:** T-tests comparing knowledge, attitude, and skill scores for visually impaired students.

Dimension		Mean	S.D.	Mean difference	95% CI: Mean Difference		Paired T-test		
					Upper	Lower	*t*	*df*	*p*
Knowledge	Pre-test	6.06	0.888	1.30	−1.208	−1.391	−27.849	456	<0.001
	Post-test	7.36	0.700						
Attitude	Pre-test	2.16	0.359	0.50	−0.469	−0.550	−24.712	456	<0.001
	Post-test	2.67	0.292						
Skill	Pre-test	3.51	0.821	0.62	−0.539	−0.685	−16.426	456	<0.001
	Post-test	4.13	0.550						

### Assessment of behavioral retention among blind students

The follow-up evaluation focused on assessing the behavioral development of visually impaired students in segregating general and face mask waste one month after the training. This assessment involved asking target schools to evaluate the sustainability of students’ waste segregation behaviors. A structured behavioral observation tool was employed, addressing various key aspects to systematically assess the outcomes and long-term impact of the training on students with visual impairments in the target schools.

Interviews revealed that visually impaired students demonstrated enthusiasm in applying their newly gained knowledge in practical settings. Notably, their interest in waste segregation was significantly heightened due to the incorporation of ‘sound’ features, which made the activity engaging and exciting for them. The use of an innovative sound-equipped bin encouraged their continued participation in waste segregation efforts, as highlighted by their responses during the interviews. One respondent noted: “The children are very fond of trash bins. They actively look for scraps like candy wrappers and pieces of paper to discard them in the bins.”

Interviews also revealed that visually impaired students demonstrated a good level of proficiency in segregating waste and disposing of face masks and other types of waste in the sound-equipped bins. Moreover, there was a noticeable improvement in their waste segregation behavior. One respondent noted: “Ever since the installation of the sound-equipped bins, the children have been segregating waste effectively. This has eliminated the need for the janitor to spend time sorting the waste afterward.”

In addition, the interviews revealed that visually impaired students exhibited a strong awareness of the importance of segregating and properly disposing of general waste. One respondent noted: “The children have learned to separate waste and are using the sound-equipped bins more frequently to dispose of face masks and other types of waste.”

The interviews also revealed that visually impaired students had gained a strong understanding of the benefits of segregating and disposing of face mask waste in the sound-equipped bins. One respondent noted: “After the training, the children are well aware of the dangers of improperly disposing of face masks and the environmental impacts this can cause.”

Finally, the interviews revealed that visually impaired students not only shared their newly acquired knowledge with their peers but they also took the initiative to promote waste segregation practices at home. One respondent commented: “The mother of a visually impaired student called the teacher to say that her child had asked her to separate waste at home, as the teacher had taught him. The mother was very pleased to see her child’s positive development and his ability to contribute to society.”

## Discussion

### Experiential interventions for teaching waste sorting to blind students with accessible tools

This study has shown that a training intervention based on experiential learning using accessible tools significantly improved blind students’ knowledge, attitudes, and skills in waste separation. Paired t-tests revealed statistically significant improvements across all dimensions: knowledge scores rose from 6.06 to 7.36 (mean difference = 1.30, p < 0.001), attitude scores increased from 2.16 to 2.67 (mean difference = 0.50, p < 0.001), and skill scores went from 3.51 to 4.13 (mean difference = 0.62, p < 0.001). These results highlight the training’s effectiveness in addressing the unique needs of visually impaired learners and promoting their active participation in sustainable waste management (Table 8). There are two key aspects to the program’s success.

Firstly, blind students effectively engaged with Kolb’s experiential learning cycle [35]. In particular, they interacted directly with waste sorting tools and sensory cues (Concrete Experience), reflected on these experiences (Reflective Observation), connected them to broader waste management principles (Abstract Conceptualization), and practiced independent waste sorting (Active Experimentation). This process fostered their comprehensive understanding and confidence in waste segregation. Teachers trained to guide blind students played a crucial role in facilitating this learning process, as further discussed below. The hands-on, reflective approach proved particularly effective, leveraging sensory-rich and real-world interactions to align with the preferred learning modalities of blind students while building critical problem-solving skills.

Secondly, the experiential learning process of blind students was facilitated by tailored interventions aligned with their sensory capabilities, consistent with the principles of Universal Design for Learning (UDL). Tools such as sound-equipped bins and waste sorting activity guides for blinds provided alternative sensory cues, addressing a significant gap in traditional waste sorting methods. Conventional tools like color-coded bins are effective for sighted individuals but fail to meet the unique learning needs of blind students [59–61]. Without appropriate and effective materials, blind students are often limited to verbal instruction in classroom settings, missing out on experiential, hands-on learning opportunities. As evidence suggests, when blind students receive materials and tools responsive to their sensory needs, their understanding and skill development improve significantly [32,62].

The tools developed in this study, such as sound-equipped bins and waste sorting activity guides for blinds, aimed to bridge these gaps by fostering independence and enhancing accessibility in environmental education, in line with the principles of Universal Design for Learning (UDL). This approach promotes inclusive learning environments while also highlighting the critical need for comprehensive research and investment in adaptive educational materials. Such innovations ensure that blind students can develop practical and sustainable skills on par with their sighted peers, further advancing equity in environmental education and waste management practices.

### The importance of teacher training for effective waste sorting education

Teacher training plays a critical role in enhancing the effectiveness of waste sorting education for blind students, particularly through experiential learning approaches. There are two key aspects of this study that highlight the importance of teacher training.

Firstly, teachers equipped with the necessary skills to teach blind students and trained to use sound-equipped bins and waste sorting activity guides for blinds acted as facilitators in all four stages of Kolb’s experiential learning cycle [35]. Effective experiential learning requires teachers with a deep understanding of the unique learning needs of visually impaired students. This relationship is pivotal, as external trainers may lack the rapport or insight necessary for addressing these specific requirements. Teachers who have established strong connections with visually impaired students are better positioned to impart essential skills and attitudes for effective waste separation. These findings align with prior research demonstrating that special education teachers with profound knowledge of their students’ needs can offer appropriate solutions [32,62]. Furthermore, teachers serve as intermediaries among stakeholders—students, parents, and policymakers—ensuring the implementation of policy through their competence and willingness to support children with special needs [63].

Secondly, teachers participating in the study played a vital role in transferring positive attitudes toward waste separation to blind students. The training program not only enhanced teachers’ technical skills with accessible tools like sound-equipped bins but also contributed to promote their environmental responsibility. The study observed modest improvements in teachers’ attitudes after the training, with scores rising from 2.61 (S.D. = 0.469) to 2.80 (S.D. = 0.359). However, the mean difference of 0.19 (95% CI: -0.12 to 0.49, t = 1.26, p > .001) was not statistically significant. This suggests that, while training can significantly improve knowledge and skills, more time or additional interventions may be required to achieve notable changes in attitudes. Importantly, teacher attitudes are critical for integrating and supporting the academic success of students with special needs. Negative perceptions, often stemming from limited resources or a lack of teaching materials, pose significant challenges [63]. Promoting positive attitudes within schools can be achieved through inclusive training and meaningful interactions with disabled students [64,65]. It has been shown that prior training provides valuable opportunities to address teachers’ concerns and positively influence their attitudes toward teaching students with special needs [66,67]. Thus, prioritizing continuous teacher training and providing adequate teaching resources can be the foundation for sustainable and inclusive waste management programs. These initiatives foster environmental stewardship among visually impaired students, creating a more equitable and effective educational environment.

## Implications

### Theoretical implications

This study advances the application of experiential learning theory by demonstrating how tailored interventions, such as sound-equipped bins and waste sorting activity guides for blinds, can effectively support environmental education for visually impaired students. By integrating sensory-based tools into Kolb’s experiential learning framework, this research bridges a gap in inclusive education and highlights the importance of adapting abstract concepts into accessible, hands-on learning experiences. The findings of the study emphasize the need for universal design principles in educational methodologies, paving the way for broader theoretical frameworks that address the diverse learning needs of students with disabilities.

### Policy implications

The findings of this study underscore the critical need for inclusive waste management policies accommodating visually impaired individuals, an aspect that has been largely overlooked in existing frameworks. In Thailand and many Global South countries, waste separation programs rely heavily on visual cues, inadvertently excluding individuals with visual impairments from meaningful participation in sustainability efforts. To bridge this gap, policymakers must integrate assistive technologies, such as sound-equipped bins, which can serve both as educational tools and practical waste management solutions, into national education policies. These adaptations do not require substantial financial investments compared to large-scale waste management infrastructure projects. Instead, embedding experiential learning for inclusive waste practices into educational systems establishes a foundation for long-term behavioral change, fostering a generation of young people actively engaged in sustainable waste management.

Developed countries, with their advanced waste management infrastructures, play a pivotal role in promoting inclusive waste policies by integrating universal design principles into existing systems. For instance, meticulous waste separation frameworks can be expanded to incorporate tactile and audio-based features, ensuring accessibility for visually impaired individuals and setting a global benchmark for inclusive waste management. This approach aligns with the United Nations Sustainable Development Goals (SDGs), reinforcing the need for accessibility in sustainability and social policies. By embedding inclusivity within waste management strategies, societies can foster equitable participation, enabling all individuals—regardless of physical ability—to contribute meaningfully to global waste reduction efforts.

### Practical implications

This study provides a framework for training educators while highlighting the need for tangible, inclusive strategies to enhance environmental responsibility among visually impaired students. Educators and school administrators should prioritize the integration of experiential learning methods alongside adaptive educational technologies, such as sound-equipped bins, which serve both as instructional tools and practical waste management solutions for visually impaired students. Currently, these sound-equipped bins have been implemented in 11 of the 16 schools for the blind nationwide. However, to maximize their impact, these devices should be made widely available in all schools for the blind and incorporated into broader sustainability programs.

Furthermore, national education frameworks should incorporate accessible waste management curricula to ensure that visually impaired students receive equitable environmental education in both developing and developed countries. Teacher training programs must emphasize inclusive pedagogical approaches, equipping educators with the competencies required to facilitate the active participation of students with disabilities. The integration of cost-effective assistive technologies within school-based sustainability initiatives offers a practical and scalable model for experiential learning in inclusive environmental education. Additionally, the promotion of best practices in accessibility-focused waste separation and the support for global knowledge-sharing initiatives can enhance sustainability efforts. Strengthening collaboration between developing and developed nations in inclusive waste education will accelerate sustainable waste management and empower visually impaired students to actively contribute to environmental stewardship.

## Limitations and conclusions

Some of the limitations of this study are related to the use of pre- and post-intervention comparisons and teacher observation to assess changes in knowledge, attitudes, and skills. Although these methods provide valuable insights, they may be undermined by self-report biases and the subjective opinions of observers. Additionally, the study lacks long-term follow-up data to determine whether the observed improvements in knowledge, attitudes, and waste-sorting behavior are sustained over time. Future studies should incorporate assessments to evaluate long-term behavioral retention. Moreover, evaluations should involve a broader range of stakeholders, including classmates, teachers, and janitors.

While the study showed an improvement in knowledge, attitudes, and waste-sorting behavior among visually impaired students in Thailand, the same method could potentially be applied in other countries with similar contexts. Hence, future research should be broader in scope, encompassing diverse geographical settings as a way to confirm and extend the applicability of the proposed intervention. Moreover, this study did not account for external variables such as policy changes, technological advancements in waste management, or cultural differences that could impact the effectiveness of the intervention in various settings. Addressing these factors in future research could provide a more nuanced understanding of the intervention’s potential scalability across different countries.

In conclusion, this study demonstrates the effectiveness of experiential learning interventions tailored to the unique needs of visually impaired students, integrating principles of universal design and experiential learning with innovative tools, such as sound-equipped bins and waste sorting activity guides for blinds to support independent waste sorting skills. Using a three-phase mixed-methods approach, including teacher training, student training, and behavioral retention evaluation, the study revealed significant improvements in students’ knowledge, attitudes, and skills, with behavioral retention observed after the intervention. This study presents a novel experiential learning model tailored specifically to blind students, integrating Kolb’s experiential learning cycle with accessible tools such as sound-equipped bins and waste sorting activity guides for blinds to address gaps in traditional waste management education. Unlike conventional methods, this approach uses sensory-responsive interventions and teacher training to foster independence and practical skills in waste separation. These findings provide valuable insights for educators, policymakers, and researchers advocating for the integration of universal design principles in education. Beyond its theoretical contributions to experiential learning in inclusive settings, the study offers a practical framework for advancing environmental responsibility, equity, and inclusivity in education, ultimately contributing to a more sustainable and inclusive global society.

## Supporting information

S1 File Original survey questionnaire for teachers used in the study. (DOCX)

S2 File Original survey questionnaire for students used in the study. (DOCX)
