# Supplementary material for: Empowering visually impaired students through innovative tools and accessible waste sorting education at the national level
Source: PLoS One. 2025 May 7;20(5):e0323171. doi: 10.1371/journal.pone.0323171 (PMC12057995; doi:10.1371/journal.pone.0323171)
Supplement: S1 File — (DOCX) [file pone.0323171.s001.docx]

**Questionnaire on teachers' knowledge, attitudes, and skills (pretest and posttest)**

**Section 1 Teachers’ knowledge about the management of general and face mask waste**

| **Waste Management Knowledge** | **Yes** | **No** |
| --- | --- | --- |
| Snack wrappers are recyclable waste. |  |  |
| Batteries are hazardous waste. |  |  |
| Plastic bottles are recyclable waste. |  |  |
| Face masks used during the COVID-19 pandemic are infectious waste. |  |  |
| Disposed face masks can decompose naturally. |  |  |
| Used face masks and COVID-19 protective equipment may be contaminated with pathogens and pose a risk to others, such as waste collectors. |  |  |
| Leakage of face mask waste into the ocean poses severe risks to marine life. |  |  |
| Face masks used during the COVID-19 pandemic can be disposed with  general waste. |  |  |

**Section 2 Teachers’ attitudes toward the management of general and face mask waste**

| **Attitude** | **Level** | | |
| --- | --- | --- | --- |
|  | **High** | **Medium** | **Low** |
| Benefits of general waste segregation for individuals with visual impairments |  |  |  |
| Benefits of segregating face mask waste for individuals with visual impairments |  |  |  |
| Satisfaction with general waste segregation for individuals with visual impairments |  |  |  |
| Satisfaction with face mask waste segregation for individuals with visual impairments |  |  |  |

**Section 3 Teachers’ skills in teaching waste segregation**

| **Skills** | **Level** | | | | |
| --- | --- | --- | --- | --- | --- |
|  | **Highest** | **High** | **Moderate** | **Low** | **Least** |
| Teaching visually impaired students to segregate and dispose of general waste. |  |  |  |  |  |
| Teaching visually impaired students to segregate and dispose of face mask waste. |  |  |  |  |  |
| Providing activities aimed at general and face mask waste segregation. |  |  |  |  |  |
| Organizing recreational activities to promote general and face mask waste segregation. |  |  |  |  |  |
